# Family Socioeconomic Status and Learning Engagement in Chinese Adolescents: The Multiple Mediating Roles of Resilience and Future Orientation

**DOI:** 10.3389/fpsyg.2021.714346

**Published:** 2021-09-02

**Authors:** Jing-Jing Chen, Ting-Na Jiang, Ming-Fei Liu

**Affiliations:** ^1^Department of Sociology, Faculty of Humanities and Social Sciences, Nanjing Forestry University, Nanjing, China; ^2^Nanjing Zhonghua High School, Nanjing, China

**Keywords:** socioeconomic status, learning engagement, resilience, future orientation, multiple mediation

## Abstract

This study explored the mediating effects of resilience and future orientation on the relationship between family socioeconomic status (SES) and learning engagement within the context of Chinese culture based on the cognitive theory of social class. A total of 1,245 junior high school students were recruited to complete anonymous questionnaires regarding the objective and subjective SES of their families, resilience, future orientation, and learning engagement. The mediating effects were tested by stepped multiple linear regression. Results indicated the following: (1) the relationships between objective and subjective SES, resilience, future orientation, and learning engagement was significantly positive; (2) resilience only mediated the relationship between subjective SES and learning engagement, whereas future orientation mediated the relationships between objective/subjective SES and learning engagement; (3) resilience and future orientation sequentially mediated the relationship between subjective SES and learning engagement. The current study contributes to a better understanding of how family SES influences adolescent academic performance from the perspective of adolescent cognitive abilities. In addition, this study provides implications for the prevention and intervention of academic performance of poor adolescents due to low SES.

## Introduction

Socioeconomic status (SES) refers to the rank or prestige of an individual in relation to others or their access to material and social resources and goods (Matthews and Gallo, 2011). It contains both subjective and objective aspects. The objective SES is used to describe the actual possession of economic and cultural resources of a family (Kraus et al., 2012), while the subjective SES is used to describe how individuals perceive their position within the social class structure (Adler et al., 2000). Numerous studies have indicated that family SES weighs heavily on the academic achievement of an individual. Kariya (2012) pointed out that adolescents of low SES in the primary and secondary school stages underperformed in learning attitudes and behaviors compared with those adolescents of middle and high SES. Other studies have also indicated that adolescents with low SES have less learning motivation and higher dropout rates (Sirin, 2005; Cheadle, 2008). Similar results have been reported in China (Fang and Feng, 2008; Sun, 2011). In contemporary Chinese society, education is still the most significant way for children of low SES to achieve upward social mobility and transform their lives (Xiong, 2017). Thus, it is of great distinction to explore the internal mechanism of how family SES influences the academic performance of adolescents under a framework with a Chinese background.

Learning engagement refers to the enthusiasm an individual has for learning and immersion (Fang et al., 2008). It not only significantly predicts the academic achievements of the individual (Li and Huang, 2010) but also significantly predicts the dropout likelihood of the individual (Furrer and Skinner, 2003; Archambault et al., 2009). Researchers think that it is difficult to examine the long-term academic achievements of an individual; they use learning engagement to describe the recent psychological state of an individual with respect to their academic activities (Hu et al., 2010b; Gu et al., 2019).

Family investment theory explains how the SES of a family affects the learning engagement of an adolescent. This theory is rooted in the economic principles of investment and builds on the notion that parents of higher SES compared with those of lower SES have greater access to financial (e.g., income), social (e.g., occupational status), and human (e.g., education) capital. According to this theory, the investment of these resources by families is associated with the successful development of children and adolescents (Conger and Donnellan, 2007) that means families with a higher SES are more likely to provide better learning conditions and material stimulation, while children from families with a low SES lack high-quality educational opportunities that can provide a motivational basis for the learning of children. Numerous empirical studies have offered supporting data for this observation (Davis-Kean, 2005; Yang and Wan, 2015; Guo et al., 2018; Li, 2018; Poon, 2020). However, when compared with research from the perspective of a family environment, few research projects have examined the relationship between family SES and academic engagement from the perspective of adolescents. The cognitive theory of social class provides this needed theoretical perspective, as it illustrates that social class contexts elicit different social cognitive patterns, which, in turn, lead to differences in the behaviors of individuals (Kraus et al., 2012; Guo et al., 2015). Modern researchers have selected two important cognitive variables regarding adolescents, namely, resilience and future orientation, as mediating variables to explore the internal mechanism of family SES on the academic engagement of adolescents.

### The Mediating Role of Resilience

Resilience refers to the ability of an individual to recover from negative experiences and flexibly adapt to a changing environment (Werner, 2000). When the level of resilience of an individual is low, it means that the motivation and ability of the individual to cope with difficulties are weaker. Therefore, resilience is thought to be an important psychological resource for individuals to cope with stressful situations (Hu and Gan, 2008). Previous studies have pointed out that the resilience of an adolescent can positively predict their academic performance (Rouse, 2001; Kotzé and Kleynhans, 2013; Kwek et al., 2013). This is due to the fact that resilience positively predicts self-motivation (Zimmerman et al., 1992), which may also improve the learning engagement of adolescents. Other research has pointed out that adolescents with a high level of resilience can mobilize more psychological resources to adjust their negative emotions on time when faced with academic pressures. These abilities lead them toward higher involvement in their academic activities (Trigueros et al., 2019).

It has also been found that an adolescent with inadequate family economic conditions has a lower level of resilience (Myers and Taylor, 1998). Some researchers believe that family SES affects resilience through the relationship between adolescents and their parents (Mackay, 2003; Gao et al., 2020; O'Gara et al., 2020). For example, parental confidence in the future of an adolescent may infuse them with high hopes, optimism, and a sense of direction, which further promotes their positive adaption during stressful situations (Gao et al., 2020). However, research has found that Chinese parents with low SES are typically so busy working that they seldom communicate with their children (Huang et al., 2019). In addition, research shows that parents with a low family SES adopt more overprotective parenting styles (Hoffman, 2003; Zhang et al., 2017). This condition may make adolescents overly dependent on their parents and cause them to develop poor frustration tolerance (Azhar et al., 2020). All of these studies indicate that lower family SES results in less-than-ideal adolescent resilience development.

We, therefore, proposed our first hypothesis; that is, resilience mediates the association between family SES and learning engagement. Thus, family SES positively predicts the resilience of an adolescent, which can further improve the learning engagement level.

### The Mediating Role of Future Orientation

Future orientation refers to the emotions and attitudes of an individual toward the future and thinking and planning for this future (Nurmi, 1991). It contains a wide range of meanings and is defined in different ways. Some researchers based it on the object of the individual looking into the future and divided the conceptual structure into the personal future and the future of society (Chen, 2017). Other studies reported that the future of the families, work, and studies of adolescents are the three most important prospects when they look into the future (Chen et al., 2013). Scholars in China construct the conceptual structure of future orientation from three dimensions, namely, (1) perceptions about the future, which refer to the frequency and time span of thoughts on the future, (2) emotions regarding the future, which can be optimistic or pessimistic, and (3) the will to execute the plan, which refers to the planning and implementation of a plan (Liu et al., 2010). Research shows that individuals who look to the future with a long-term perspective or with clearer goals will more accurately be able to predict their learning engagement through delayed gratification or the adoption of more learning strategies (Pang et al., 2014; Carvalho, 2015; Du and Lv, 2017).

Future orientation is a process of continuous construction under a specific social and cultural background (Massey et al., 2008), and the family environment has the most important influence on it (Malmberg, 2001). Research shows that a lack of familial economic and cultural resources restricts the development of adolescent future imagination (Zhao, 2018) and makes it difficult for adolescents to see and plan for the future (Wang and Ma, 2019). In Chinese rural areas, the notion that “study is useless” still prevails, suggesting that low family SES is closely related to low educational expectations. These low educational expectations lead to higher dropout rates (Chai and Lv, 2017).

Therefore, we proposed a second hypothesis; that is, lower familial SES causes lower levels of future orientations and, thus, is a negative predictor of the academic engagement of an adolescent.

### The Multiple Mediation Model

Studies have verified resilience as positively correlated with future orientation (Seginer, 2008) and positive expectations for the future (Wyman et al., 1992). Studies have also pointed out that positive future orientation is one of the important factors for individuals to overcome obstacles and obtain good adaptations (Li et al., 2015; Hatala et al., 2017). When faced with stressful situations, individuals use protective coping factors to manage risks and maintain physical and mental balance (Shen, 2010). These protective factors include the planning and optimism an individual has for the future (Hu and Gan, 2008; Hu et al., 2010a). Therefore, when faced with academic pressure, adolescents with a high level of resilience may manage their obstacles by setting goals, formulating plans, and implementing solutions. These actions result in a higher level of academic engagement.

Thus, we proposed a third hypothesis: the relationship between family SES and learning engagement may be sequentially mediated by the resilience and future orientation.

### The Present Study

**Figure 1:**
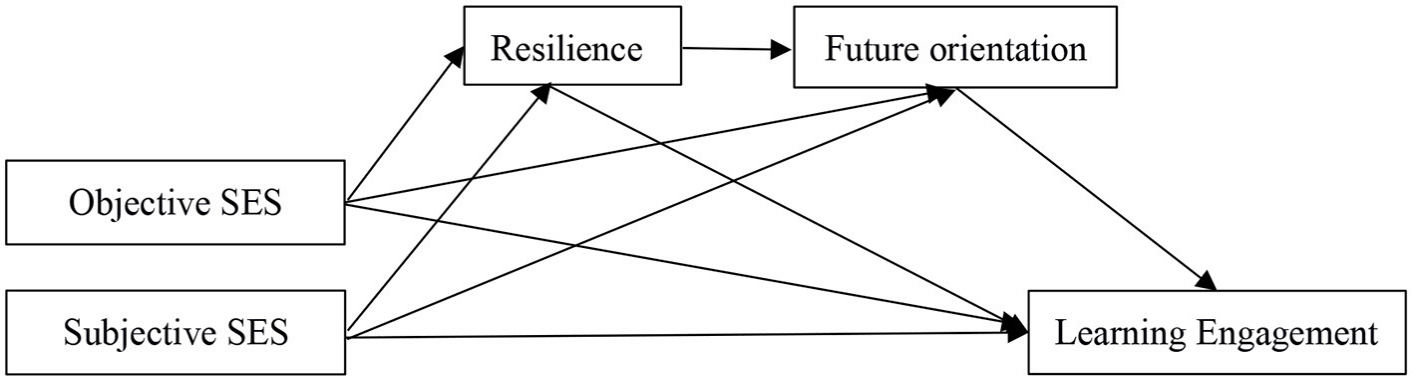
Conceptual model. SES, socioeconomic status.

In summary, this study aimed to verify the mediating effect of resilience and future orientation with regard to the relationship between family SES and adolescent academic engagement. The results of the present study not only provide theoretical knowledge to improve student academic performance but can also provide practical guidance for school education and teaching. The hypothetical model is shown in Figure 1.

## Methods

### Participants

The convenience sampling method was used to select the schools studied in this research. Two public junior high schools located separately, from either an urban or rural area in Jiangsu Province, assisted in this investigation. A questionnaire survey was conducted in the first to the third grades using the cluster sampling method and based on the class as a unit. In China, due to the dual structure of urban and rural areas, compared with rural areas, parents in urban areas, as a whole, have higher educational and occupational levels, more diverse types of occupations, and stronger economic abilities (Feng and Xiao, 2014). Meanwhile, rural adolescents, as a whole, have lower educational expectations, higher dropout rates, and worse academic achievements (Li et al., 2020). The participants in this study were composed of people living in urban and rural areas, which means that the sample data was representative and represented the large internal differences in family SES and academic performance. A total of 1,300 questionnaires were distributed, with 1,245 retrieved (95.77% recovery rate). Fraudulent questionnaires were eliminated. A total of 1,156 valid questionnaires were used for the data analysis. Among the respondents, 582 were boys (50.3%) and 551 were girls (47.7%). A total of 23 questionnaires lacked gender information. There were 303 (26.2%) first-grade students (aged 12–14 years), 505 (43.7%) second-grade students (aged 13–15 years), and 344 (29.8%) third-grade students (aged 14–16 years). A small number (4) of questionnaires lacked grade information. Respondents were an average of 13.78 years old and the standard deviation was 0.95.

### Measures

#### Subjective and Objective SES

A study by Matthews and Gallo (2011) shows that objective SES is measured from two aspects: parental educational level and family income. In this study, for the measurement of parental educational level, six options ranging from “primary school or below” to “post-graduate (master or doctoral)” were set, and points from 1 to 6 were assigned. Monthly household income was collected, and we confirmed the average monthly income in the survey city. Subsequently, 10 option levels ranging from “below 2,000 yuan” to “above 10,000 yuan” were set, and they were assigned a value from 1 to 10 points. After standardizing the scores of the two measurement indicators, the sum of the scores was taken as the index of objective SES. Higher scores meant a higher level of objective SES.

Subjective SES was measured using a self-edited question: “Compared to the local situation, what do you think of your family's economic situation?” A total of 5 options from “very poor” to “very good” were set, and they were assigned a value from 1 to 5 points. Higher scores meant a higher level of subjective SES.

#### Resilience

Resilience was measured using the Resilience Scale (RS; Wagnild and Young, 1993), which was revised by Hu and Gan (2008) to contextually account for Chinese culture. This scale includes 27 items (e.g., “Failure always makes me feel discouraged”) and assessed two dimensions: personal strength (15 items) and support (12 items). A 5-point scoring system was used, and values between 1 and 5 points were assigned from “completely inconsistent” to “completely consistent.” Higher scores meant a higher level of resilience. In this study, Cronbach's α of the two sub-questionnaires and the overall questionnaire ranged from 0.78 to 0.86.

#### Future Orientation

Future orientation was measured on a Chinese version of the Future Orientation Scale, which was invented by Liu et al. (2011). It contains 31 items (e.g., “I often think about things to do in the future”) and assesses three dimensions: perceptions about the future (9 items), emotions for the future (10 items), and the will to execute a plan (12 items). Participants rated each item on a 5-point scale ranging from “completely inconsistent” to “completely consistent.” The average of the 31 items was calculated, with a higher score indicating a greater capacity to perceive the future as expansive and optimistic. Cronbach's α of the three sub-questionnaires and the overall questionnaire ranged from 0.85 to 0.91 in this study.

#### Learning Engagement

The Learning Engagement Scale was invented by Fang et al. (2008). It assesses three dimensions: vitality (6 items), dedication (5 items), and focus (6 items), with a total of 17 items. A 5-point scoring system was used, and values of 1 to 5 points were assigned from “completely inconsistent” to “completely consistent.” The average of the 17 items was calculated, with a higher score indicating a higher level of learning engagement. Cronbach's α coefficients of the three sub-questionnaires and the overall questionnaire ranged from 0.8 to 0.92 in this study.

See Appendix for the contents of all the scales.

### Procedures and Data Analysis

The contents of the questionnaire were first sent to school leaders for an advanced review. After obtaining permission to conduct the survey, the school notified parents and students of the content of the survey and received permission to proceed. The survey was conducted using a part of the classroom time of students. Before filling in the questionnaire, participants were informed of the anonymity of the questionnaire and matters unrelated to school academic performance. All the questionnaires were collected upon completion.

Analyses were performed using SPSS 19.0 and the PROCESS macro for SPSS (Hayes, 2013). First, we conducted the analyses of descriptive statistics and a Pearson's correlation analysis to have a preliminary overview of the study variables. Next, we used PROCESS 3.3 to run the serial mediation analyses using Model 6. Direct and indirect effects were estimated using the bias-corrected non-parametric bootstrapping techniques of Preacher and Hayes with 5000 bootstrap samples (Preacher and Hayes, 2004). Research showed (Settanni et al., 2018) the existence of mediation effects that were further evaluated using 95% bias-corrected CIs. When the CIs did not contain a zero, then these effects were considered statistically significant.

## Result

### Preliminary Analysis

Descriptive statistics and the correlation matrix of variables are shown in Table 1. Objective SES was significantly and positively associated with subjective SES. Both objective SES and subjective SES were significantly and positively associated with resilience, future orientation, and learning engagement. Resilience was significantly and positively associated with future orientation and learning engagement. Future orientation was significantly and positively associated with learning engagement.

**Table 1:** Descriptive statistics and inter-correlations between study variables (*N* = 1,245).

	***M***	***SD***	**1**	**2**	**3**	**4**	**5**
1 Objective SES	0.03	2.32	1	0.34^**^	0.12^**^	0.20^**^	0.15^**^
2 Subjective SES	3.21	0.64		1	0.21^**^	0.25^**^	0.21^**^
3 Resilience	3.50	0.61			1	0.61^**^	0.53^**^
4 Future Orientation	3.46	0.63				1	0.65^**^
5 Learning engagement	3.34	0.79					1

** *p < 0.01*.

### Mediation Effect Analysis

The PROCESS macro was used to examine the multiple mediating roles of resilience and future orientation in the relationships between objective/subjective SES and learning engagement. Table 2 shows that objective SES was not significantly associated with resilience, but subjective SES was positively associated with the same variable (β = 0.19, *p* < 0.001). Resilience was also positively associated with learning engagement (β = 0.2, *p* < 0.001). Meanwhile, both objective SES (β = 0.09, *p* < 0.001) and subjective SES (β = 0.1, *p* < 0.01) were positively associated with future orientation, which was, in turn, positively associated with learning engagement (β = 0.53, *p* < 0.001). Resilience also showed a positive association with future orientation (β = 0.58, *p* < 0.001). Furthermore, both objective SES (β = 0.1, *p* < 0.05) and subjective SES (β = 0.19, *p* < 0.001) were positively related to learning engagement.

**Table 2:** Multiple mediation analysis results.

**Regression model**		**Goodness-of-fit indices**			**Regression coefficient and significance**			
**Outcome variable**	**Predictor variable**	***R***	***R*^**2**^**	***F***	**β**	***t***	**95%CI lower limit**	**95%CI upper limit**
Resilience	Objective SES	0.23	0.05	20.53^***^	0.06	1.66	−0.01	0.04
	Subjective SES				0.19	5.13^***^	0.12	0.26
FO	Objective SES	0.64	0.40	169.25^***^	0.09	3.00^***^	0.01	0.04
	Subjective SES				0.10	3.36^**^	0.04	0.16
	Resilience				0.58	20.04^***^	0.52	0.64
LE	Objective SES	0.68	0.47	165.30^***^	0.02	0.01	−0.01	0.02
	Subjective SES				0.04	1.12	−0.03	0.11
	Resilience				0.20	5.78^***^	0.16	0.33
	FO				0.53	15.45^***^	0.59	0.76
LE	Objective SES	0.24	0.06	23.30^***^	0.10	2.58^*^	0.01	0.06
	Subjective SES				0.19	4.91^***^	0.14	0.32

*FO, future orientation; LE, learning engagement*.

* *p < 0.05*,

** *p < 0.01, and*

*** *p < 0.001*.

The results of the bootstrap analysis are shown in Table 3. The analysis results showed that the total mediating effect of objective SES on learning engagement was significant [Bootstrap 95% CI (0.01, 0.04)], which accounted for 81.91% of the total effects. Specifically, the effect of the path “Objective SES → Resilience → Learning engagement” was 0.004 and accounted for 12.58% of the total effects [Bootstrap 95% CI (−0, 0.01)]. The effect of the path “Objective SES → Future orientation → Learning engagement” was 0.016 and accounted for 49.39% of the total effects [Bootstrap 95% CI (0.01, 0.03)]. The effect of the path “Objective SES → Resilience → Future orientation → Learning engagement” was 0.007 and accounted for 19.94% of the total effects [Bootstrap 95%CI (−0.00, 0.01)]. Both the 95% CIs of the paths “Objective SES → Resilience → Learning engagement” and “Objective SES → Resilience → Future orientation → Learning engagement” included 0, which suggested that the mediating effect of resilience and the sequentially mediating effect were not significant. Only future orientation significantly mediated the relationship between objective SES and learning engagement.

**Table 3:** Bootstrap analysis of multiple mediation effects.

	**Effect size**	**Bootstrap SE**	**Bootstrap 95%CI**	**Percentage of total effects**
The total mediating effects	0.027	0.008	[0.01, 0.04]	81.91%
Objective SES → Resilience → LE	0.004	0.003	[−0.00, 0.01]	12.58%
Objective SES → FO → LE	0.016	0.005	[0.01, 0.03]	49.39%
Objective SES → Resilience → FO → LE	0.007	0.004	[−0.00, 0.01]	19.94%
The total mediating effects	0.189	0.032	[0.13, 0.25]	82.52%
Subjective SES → Resilience → LE	0.047	0.013	[0.02, 0.08]	20.52%
Subjective SES → FO → LE	0.068	0.022	[0.03, 0.11]	29.69%
Subjective SES → Resilience → FO → LE	0.074	0.016	[0.04, 0.11]	32.31%

*FO, future orientation; LE, learning engagement*.

Meanwhile, the analysis results showed that the total mediating effect of subjective SES on learning engagement was significant [Bootstrap 95%CI (0.13, 0.25)] and accounted for 82.52% of the total effects. Specifically, the effect of the path “Subjective SES → Resilience → Learning engagement” was 0.047; it accounted for 20.52% of the total effects. The effect of the path “Subjective SES → Future orientation → Learning engagement” was 0.068, accounting for 29.69% of the total effects. The effect of the path “Subjective SES → Resilience → Future orientation → Learning engagement” was 0.074, which accounted for 32.31% of the total effects. The 95% CIs of all the paths did not include 0, suggesting that resilience and future orientation mediated the relationship between subjective SES and learning engagement not only in parallel but also sequentially.

## Discussion

The relationship between family SES and educational attainment is an important indicator to measure educational equity (Li and Qiu, 2016). The gap between the rich and the poor in China has been widening in recent years, and the socioeconomic conditions of families are playing an increasingly important role in the education of children (Li, 2016). In this context, and based on the cognitive theory of social class, this study verified the mediating roles of resilience and future orientation between family SES (objective/subjective) and learning engagement.

### The Correlation Between the Variables

The results of the correlation analysis showed that there was no high correlation between subjective and objective SES (*r* = 0.34), which was consistent with the conclusions of previous studies (Kraus and Stephens, 2012). Previous results suggested that, due to certain reasons such as survival anxiety, the subjective evaluations of family SES among Chinese people are generally lower than the objective evaluation (Fan and Chen, 2015). Regardless of the subjective or objective SES, our results showed that both of them were significantly and positively correlated with resilience, future orientation, and learning engagement. In addition, learning engagement has a moderate correlation with resilience and future orientation; this indicates that both objective and subjective SES is closely related to learning engagement, thus providing a theoretical basis for the further analysis of the shown mediating effect.

### The Mediating Effect of Resilience

Even though studies have shown that individuals of lower SES are more likely to adopt negative coping styles in stressful situations (Leyva et al., 2020), in this analysis, objective SES had no significant effect on resilience. This conclusion may be explained by the Chinese cultural environment specifically. One typically traditional Chinese cultural idea is that *children from poor families take charge early*, meaning that families living in poverty raise children who show more tenacity and maturity when facing the obstacles in their lives. Empirical research has supported this idea, and it can be used to verify that rural adolescents in the middle and lower classes of Chinese society have stronger adjustment abilities and show stronger resilience in the face of adversity (Wu et al., 2011). In addition, previous studies have highlighted the fact that parents of low family SES adopt more punitive and authoritarian parenting styles (Hoffman, 2003; Kaiser et al., 2019). Such conditions may make adolescents feel low self-efficacy (Li et al., 2010) and cause them to develop poor resilience (Azhar et al., 2020). However, the results of similar research in the context of Chinese culture showed that punitive and authoritarian parenting styles do not show any adverse effects on the development of adolescents (Chao, 2001). Some Chinese scholars have pointed out that this is because many Chinese adolescents, under the influence of traditional cultural concepts, tend to interpret the strictness of their parents as love (Qu et al., 2016). Therefore, our results showed that objective family SES did not have a negative impact on resilience, which could be due to the fact that family SES has different effects on resilience through the mediating effect of parental rearing styles. Since the relationship between objective SES and resilience is still unclear, the mediating effect of resilience on the relationship between objective SES and learning engagement has been found to be insignificant.

Further results showed that resilience has a significant mediating effect on the relationship between subjective SES and learning engagement. Existing research pointed out that, although the formation of subjective SES is related to objective SES to a certain extent, they are primarily drawn from social comparisons; that is, how individuals evaluate their economic status in comparison with others also has an effect (Gao, 2018). The results of social comparisons can result in a cognitive evaluation that one is superior or inferior to others. This is likely to trigger the emotional responses of an individual and, thus, further influence the judgment and behavior of the individual (Gong and Zhang, 2020). These results suggest that the higher the subjective SES, the greater the chance that adolescents may achieve a similar superiority of their family in terms of economic and cultural resources. These benefits enable them to have more psychological resources for self-adjustment when facing situations with academic pressure.

### The Mediating Effect of Future Orientation

The results showed that future orientation significantly mediates the relationship between objective SES and learning engagement. Therefore, a higher objective SES predicts a higher level of future orientation development and, in turn, further improves the level of learning engagement. These conclusions are consistent with previous studies, which showed that parents from higher SES families possessed higher expectations for the future of their children, with these expectations eventually being internalized by those children (Kanomata, 2014; Li et al., 2016). High expectations for the future will prompt children to look forward to and plan for their futures with a long-term perspective. This is an additional incentive for adolescents to actively participate in academic activities (Yang, 2006; Wang and Ma, 2019). Other information suggested that the social networks of individuals from low SES backgrounds were highly homogeneous (Zhang and Ye, 2010), meaning that adolescents from families of lower SES have fewer opportunities to interact with people engaged in diversified occupations, which often limits their visions of the future and their recognition of educational value, thus negatively affecting their scholastic learning motivation (Gu et al., 2019).

On the contrary, other data highlighted that future orientation played a significant mediating role in the relationship between subjective SES and learning engagement. Some studies revealed that, at an early age, adolescents from lower-SES families recognized that the financial difficulties of the family would limit their ways out of such financial difficulties in the future; this often prevents them from planning or setting goals for their futures. They tend to live in the present (Chen, 2017), which results in low motivation for academic activities (Chai and Lv, 2017).

### The Multiple Mediation Model

Our data revealed that subjective SES could also influence learning engagement through serial mediation using resilience and future orientation. This suggests that, if adolescents have an awareness of the resource advantage of their families, their level of resilience will increase; meaning that adolescents are then prompted to adopt more adaptive behaviors, such as setting goals and implementing plans, when facing academic stressful situations. These conditions have the effect of further improving their level of learning engagement.

### Objective/Subjective SES and Learning Engagement

Our data showed that subjective and objective SES have different influence mechanisms on the learning engagement of adolescents. Objective family economic and cultural resources improve the imagination and motivation of adolescents for the future by providing them with more opportunities to experience cultural activities, which further enhances the learning engagement of these adolescents. On the other hand, subjective family SES contributes to learning engagement with more paths for influence. Previous studies have illustrated that the objective economic and cultural resources of a family do not directly affect the adolescent learning engagement of their children, but the values or thinking patterns formed in their specific SES are key factors that affect the academic performance of adolescents (Chen and Xu, 2020). Our data suggested that objective SES also influences academic performance by affecting the development of the cognitive abilities of adolescents to some extent, but the subjective SES of adolescents has a greater impact on their academic performance.

## Implications and Limitations

The findings of this study provided practical insights into how to improve the academic performance of adolescents of low SES. For adolescents of middle and lower SES, school teachers or parents should focus on the cultivation and improvement of their resilience and simultaneously support and encourage students to explore and plan their futures to improve or positively affect their scholastic learning engagement. In addition, social comparisons between family economic capacities among adolescents should be avoided as much as possible to prevent adolescents of low SES from forming negative evaluations of their family environments. These negative evaluations are likely to further erode their academic performance.

The limitations of this study should be acknowledged. First, the present study used a cross-sectional design, which was not conducive to clarifying the causal relationship between variables. Longitudinal tracking is needed to further examine the dynamic relationship between subjective and objective social class, resilience, future orientation, and learning engagement. Second, this study used observed variables in statistical analysis. The results may have been more accurate if latent variables were included. Third, when measuring objective SES, this study referred to previous studies and collected data utilizing self-reporting. However, since the junior high school students may not have been very clear about their family income, such a survey method may have affected the accuracy of the survey results. Therefore, in the future, adjustments should be made in the design of this item, or parents should assist in the participation of students in the survey to improve the accuracy of the survey results.

## Conclusion

Research conclusions are summarized as follows: (1) The correlation between subjective and objective SES, resilience, future orientation, and learning engagement were significantly positive. (2) The objective family SES and subjective family SES had different influential mechanisms on the learning engagement of adolescents within the context of Chinese culture. This means that objective SES only affected learning engagement through future orientation, while subjective family SES not only affected learning engagement through resilience and future orientation but also the chain mediating effects of resilience and future orientation.

Different from previous studies that explored the mechanism of family SES on the academic performance of adolescents from the perspective of family environmental factors, and based on the cognitive theory of social class, this study clarified that both subjective and objective family SES can predict the academic performance of adolescents through the effects SES has on their cognitive abilities.

## Data Availability Statement

The original contributions presented in the study are included in the article, further inquiries can be directed to the corresponding author.

## Ethics Statement

Ethical review and approval was not required for the study on human participants in accordance with the local legislation and institutional requirements. Written informed consent to participate in this study was provided by the participants' legal guardian/next of kin.

## Author Contributions

J-JC designed the framework of this research, analyzed the data, and wrote the manuscript. T-NJ assisted in improving the framework of this research. M-FL assisted in the investigation. All authors contributed to the article and approved the submitted version.

## Conflict of Interest

The authors declare that the research was conducted in the absence of any commercial or financial relationships that could be construed as a potential conflict of interest.

## Publisher's Note

All claims expressed in this article are solely those of the authors and do not necessarily represent those of their affiliated organizations, or those of the publisher, the editors and the reviewers. Any product that may be evaluated in this article, or claim that may be made by its manufacturer, is not guaranteed or endorsed by the publisher.
