# Supplementary material for: Family Socioeconomic Status and Learning Engagement in Chinese Adolescents: The Multiple Mediating Roles of Resilience and Future Orientation
Source: Front Psychol. 2021 Sep 2;12:714346. doi: 10.3389/fpsyg.2021.714346 (PMC8444631; doi:10.3389/fpsyg.2021.714346)
Supplement: Supplementary file 1 [file Data_Sheet_1.doc]

**Appendix**

**Resilience Scale** (Hu & Gan, 2008)

1. Failure always makes me feel discouraged.

2. It is difficult for me to control my unpleasant emotions.

3. I have a clear goal in my life.

4. I will generally be more mature and experienced after experiencing setbacks.

5. Failure and setbacks will make me doubt my abilities.

6. When I encounter unpleasant things, I can't find one to talk to.

7. I have a friend of the same age who can tell him/her about my troubles.

8. My parents respect my opinion very much.

9. When I encounter troubles and need help, I don't know whom to rely on.

10. I think the process of things can help people grow more than the results.

11. When faced with difficulties, I usually make a plan and solution.

12. I am used to holding things in my heart instead of telling others.

13. I think adversity has an motivating effect on people.

14. Adversity is sometimes a kind of help to growth.

15. Parents always like to interfere with my thoughts.

16. No one listens to what I say when I’m at home.

17. My parents have few confidence and spiritual support for me.

18. I will take the initiative to talk to others when I have troubles.

19. My parents never scold me.

20. When facing difficulties, I will concentrate all my energy.

21. It usually takes a long time for me to forget unpleasant things.

22. My parents always encourage me to go all out.

23. I am able to adjust my emotions very well in a short period of time.

24. I will set goals for myself to push myself forward.

25. I think everything has its positive side.

26. I don't want to tell others when I am in a bad mood.

27. My mood fluctuates a lot, and it's easy to fluctuate.

**Future Orientation Scale** (Liu, Huang, & Bi, 2011)

1. I am full of confidence in the future.

2. I often make plans based on my actual situation.

3. I can't persevere in what I want to do.

4. I rarely think about the future.

5. I am full of expectations for the future.

6. In the process of completing the task, I can resist all kinds of temptations from the outside world.

7. I feel a little worried when thinking about the future.

8. Looking forward to the future makes me feel comfortable.

9. I often finish the task at the last moment.

10. The beautiful imagination of the future often comes to my mind.

11. There are too many uncertainties in the future, and I am afraid of going into the future.

12. Before doing things, I will make a detailed schedule.

13. I often think about some things to do in the future.

14. I will implement the plan carefully.

15. I can imagine my life ten years from now.

16. In my free time, I like to imagine the future.

17. I have great enthusiasm for the future life.

18. My plan is often abandoned halfway for various reasons.

19. I often outline my future life in my heart.

20. I often worry about my future.

21. I often think about what to do in the next five years.

22. I often think about my future life journey.

23 I like planned learning.

24. I can imagine what I will be like in a few years.

25. The uncertainty of the future makes me nervous.

26. I believe that I have the ability to create a better future.

27. I have been preparing for my future.

28. Once I decide what to do, I think about how to accomplish it.

29. We should plan our daily life well in advance.

30. I like to set goals for myself and work hard for them.

31. Facing the future, I am a little at a loss.

**Learning Engagement Scale** (Fang, Shi, & Zhang, 2008)

1. As soon as I get up in the morning, I'm ready to study.

2. I find learning challenging.

3. When studying, I often forget everything around me.

4. I feel energetic while studying.

5. Learning can inspire me.

6. When studying, I often feel that time passes quickly.

7. Even if the study is not smooth, I am not discouraged and can persevere.

8. I am passionate about learning.

9. When studying, I just concentrate on it.

10. I can continue to study for a long time without having to rest in between.

11. It is difficult for me to let go of my learning.

12. When studying, even if I am mentally tired, I can recover quickly.

13. I am proud of my studies.

14. I am immersed in learning.

15. When I study, I am strong and motivated.

16. My study purpose is clear, and I find it meaningful.

17. I feel very happy when I devote myself to studying.
